# Meteorological Table

**Published:** 1867-03

**Authors:** J. G. Westmoreland

**Affiliations:** Smithsonian Institute, Feb. 1867, at Atlanta, Ga.


					﻿6
' a H	.a . .
So	rX°2 . -^!z	.	• • -M	fc •S,H“ -H
U	” rna2 ai^i tnaiai^^ai'Z aiainiairn	Z ni
gs 'ce
_____________________________:-------------------------
Q . . ...
gs S a	• • . . .«*
So	25	ct	.	.
-h q	> rH ad cd cd cd 25 cd Z cd cd Z	Z cd cd cd Z	cd
<$ 2
If-----------------------------------------------------------------
• ...
cn	a	£■ ,®	. -a ..fe\. fcfe . a
■io f-r-i	-4 qq cd . oQ	cd , H a; cd H*H .HHH
»	00 Z Z Z® «i td 02 02 W 02
e cc ____________
.§ O	■ ---
e I
1
S t?	53 ooicciocaiaioocooooiaiainBiacoiaicnoioo
■~ M	.	rHrH rH rHrH rH rd	rH
g <!	*
oq e-i	«>
o---------------------------------------------------------------
*=> K
,S	02
+"> 5	S	3’ moocoomocoowoioiadomiamooiaoiaoiao
r-2	M	rH r< r* H rH	rH rH	rH rH rH
H	S	r
J	Q
B_______________________________________________________________
'S
e
~§	8	moiaoioocowoooooiaioioioioioooiooooia®
g	r-l rH rH	rH rH rH	rH	rH rH rH rH
gS	°
is
r _____________________________________________________________
£t
. 0§	Z	S	to	o «o
■ • 2
.	p3	5
S .
<> *§	U	*’	cqoq^ocno^^tNomoooicooiowoococco-NOOto
g	p/	CO XO AO >Q AO XO XO XO 03 03	«© XO *D CD CD XO © fc- CD CO AO b- b- CD <D 1Q
§ i<	H	cd_________________________________,________________
S •	X
i£>	$	S	O CO eq CO CQ O rH 03 «© <D rH CO OeiOCO^CO-Hb-OOlO wo^
HO	o	gq	CO XO XO XO Tt< -*f xO XO XO tQ CO •’T XO XO *© aO aO XO <X> *D *0 XO 1O b- b- CD CD AO
00 I	§	tH
2*	P4--------------------------------------------------------
a 2	°>
•£> O	kh	cj o eq © co b- oo o r-< o © oo m © co oo -h t-a m< co eq oo o cd
CO	□	<4 XO XQ AO r? aO XO XQ CO rf< XO CD AO tT t$< *D CD CD CO XO XO CD b- XO XQ
£> u ________ ^>___________________________________________________
.	s	X'	X	3
J§ £>	,	CDCOiQCDCDCD X**0	X	^*©05000	O
o g * gggggga%ggsgsgsg§§gsggsg^8a
si S H	v.
£H	CD CD XO 1O xo CD	t— b-	4^	CO C5 Ci Ci Di Oi
ciciciciciciciCicicioo©>OOOo®OC’OCicicocicicioi
rH QqQqQqoqcqeqcqoqaqcqcocooqcococococococococqQqQqQqcqaqeq
-S o	«	-------T----------------------------------------------
g^>	H	3
g CO	M	.	«o io m ® ir «o >r t-. ^»cs	oo ci o o> at ca
SO .	-"I ci CD Ci Ci ci ci ci ci ci ci ci o o ci O O © Ci O O O Ci ci 00 CO ci ci ci
ng	eq eq eq eq eq oq eq eq ox eq eq co co oi co co co ex co co co eq cq eq eq eq eq eq
»ay of Month. | -10”3'*1®®
				

